# Detection of Oral Human Papillomavirus in HIV-Positive Men Who Have Sex with Men 3 Years after Baseline: A Follow Up Cross-Sectional Study

**DOI:** 10.1371/journal.pone.0102138

**Published:** 2014-07-17

**Authors:** Jason J. Ong, Tim R. H. Read, Lenka A. Vodstrcil, Sandra Walker, Marcus Chen, Catriona S. Bradshaw, Suzanne M. Garland, Sepehr N. Tabrizi, Alyssa Cornall, Andrew Grulich, Jane Hocking, Christopher K. Fairley

**Affiliations:** 1 Melbourne School of Population and Global Health, University of Melbourne Parkville, Victoria, Australia; 2 Melbourne Sexual Health Centre, Carlton, Victoria, Australia; 3 Central Clinical School, Monash University, Clayton, Victoria, Australia; 4 Department of Obstetrics and Gynaecology, University of Melbourne, Parkville, Victoria, Australia; 5 Regional HPV Lab Net Reference Laboratory, Department of Microbiology and Infectious Diseases, The Royal Women’s Hospital, Parkville, Victoria, Australia; 6 Murdoch Childrens Research Institute, Parkville, Victoria, Australia; 7 Kirby Institute, University of New South Wales, Darlinghurst, New South Wales, Australia; Kagoshima University Graduate School of Medical and Dental Sciences, Japan

## Abstract

**Background:**

Human papillomavirus (HPV) is a causative agent in oropharyngeal squamous cell carcinoma. The natural history of oral HPV in HIV-positive men who have sex with men (MSM) is unclear.

**Methods:**

Detection of oral human papillomavirus in 173 HIV-positive MSM using oral rinse samples 3 years apart was investigated. HPV DNA was detected by polymerase chain reaction, and genotyped by Roche Linear Array.

**Results:**

Of 173 men tested in 2010, 30 had at least one HPV genotype (17%, 95% CI: 12–23), 15 at least one hr-HPV (9%, 95% CI: 5–14) and 8 had HPV 16 (5%, 95% CI: 2–9) detected. In 2013, 33 had at least one HPV genotype (19%, 95% CI: 14–26), 20 had at least one hr-HPV (12%, 95% CI: 7–17) and 7 had HPV 16 (4%, 95% CI: 2–8) detected. Of 30 men at baseline (2010) with any HPV detected, 14 (47%, 95% CI: 28–66) had at least one persistent genotype. Of the 15 men in 2010 with high risk (hr-) HPV, 6 men (40%, 95% CI: 16–68) had at least one persistent hr-HPV genotype. The incidence rate of detection of at least one new HPV genotype was 4.8 per 100 person years (95% CI: 3.1–7.0), of at least one hr-HPV genotype was 3.2 per 100 person years (95% CI: 1.8–5.1) and of HPV 16 was 0.8 per 100 person years (95% CI: 0.2–2.0). The clearance rate was 14.9 per 100 person years (95% CI: 8.2–24.2) for any HPV, 18.2 per 100 person years (95% CI: 8.2–32.7) for hr-HPV and 17.4 per 100 person years (95% CI: 5.0–38.8) for HPV-16. Persistent HPV detection was associated with duration of HIV (OR 1.13 (per additional year), 95% CI: 1.00–1.26) and tonsillectomy (OR 8.17, 95% CI: 1.30–51.40).

**Conclusion:**

The same oral HPV genotype was detected again after 3 years in nearly half of HIV-positive men who have sex with men.

## Introduction

Human papillomavirus (HPV) causes a significant proportion of oropharyngeal cancers, most commonly in the lingual and palatine tonsils or base of tongue. There has been an increase in oropharyngeal cancer incidence in younger individuals and the proportion of these tumours associated with HPV continues to rise [1]. Specific genotypes of HPV identified as high-risk [2] have been shown to be more likely to cause malignancy due to modulation of two important tumor suppressors, p53 and Rb by the HPV oncoproteins, E6 and E7 [3]. It is estimated that high risk (hr-) HPV DNA is detected in more than half of oropharyngeal cancers, and by far the most common genotype is HPV-16 [4]. It has been hypothesized that the persistence of hr-HPV infection is a prerequisite for oropharyngeal cancer [5].

Understanding the natural history of oral HPV is important for determining the significance of HPV detection. The prevalence of oral HPV detection is higher in HIV positive than in HIV negative people [6] and several natural history studies have been published but none have provided follow up data beyond a median of two years [7]. Sexual behaviors and HIV status are associated with oral HPV detection [6] but the factors associated with risk of persistent oral HPV have not been identified.

We previously measured oral HPV detection among HIV positive men who have sex with men (MSM) [8] and report here the results of a follow up study of this cohort that aimed to determine the persistence of HPV detection three years later among those participants who were previously positive.

## Methods

This was a prospective study of MSM attending the Melbourne Sexual Health Centre (MSHC) HIV clinic who participated in an oral HPV sampling and prevalence study in 2010 [8]. If these men attended MSHC again between 4^th^ Dec 2012 and 6^th^ Aug 2013, they were invited to participate in this follow up study and written informed consent was sought. At the time of their clinic visit, participants provided an oral rinse sample (ORS) that involved swishing and gargling 20 mL of sterile saline in the oral cavity for 10–20 seconds and then spitting into a specimen cup. This was the same method used for collection of samples in 2010. A questionnaire was completed by participants that asked about basic demographics, oral hygiene habits, smoking status and sexual practices (Appendix S1).

ORS samples were centrifuged for 15 minutes at 4,600 *g* and subsequently resuspended in 800 µl of PBS. An aliquot of 200 µl was extracted by the automated MagNA Pure 96 isolation and purification system (Roche Molecular Systems) using DNA and Viral NA Small Volume isolation kit. Following nucleic acid isolation, all samples were initially assessed for DNA adequacy with a quantitative PCR for a 260 bp fragment of the human beta-globin gene using 10 pmol each of β-globin primers GH20 [5′-GAAGAGCCAAGGACAGGTAC-3′] and PCO4 [5′-CAACTTCATCCACGTTCACC-3′] and 2 pmol of adapted probe PCO3 [5′-6-FAM-ACACAACTGTGTTCACTAGC-TAM-3′] [9]. Samples which were HPV-positive by PCR-ELISA [10] were subsequently genotyped by LINEAR ARRAY (LA) HPV Genotyping Test (Roche Diagnostics) [11], with modification as described previously [12]. Samples which were HPV-positive by PCR-ELISA but negative by LA were amplified using the more sensitive HPV SPF10-LiPA 25 assay version 1 (Labo Bio-medical Products BV, Rijswijk, The Netherlands). Definitions of hr-HPV genotypes were made according to recent International Agency for Research on Cancer nomenclature [2].

### Data analysis

Statistical analyses were performed using STATA (*Stata Statistical Software: Release 13.* College Station, TX). For each participant, data from both the 2010 study and the current study were analysed and compared. Proportions of individuals infected with HPV types were calculated for type-specific persistence and the detection of new HPV genotypes. Persistent HPV was defined as detection of at least one of the same HPV genotype at both study timepoints with a mean difference of 2.9 years. A newly acquired detection (incident detection) was defined in this study as at least one new HPV genotype in 2013 in a person who was negative for that HPV genotype in 2010. A cleared infection was defined as the lack of detection in 2013 of at least one specific HPV genotype that was detected in 2010. From this, incidence and clearance rates of at least one HPV type per 100 person years and 95% confidence intervals were calculated for individuals. Prevalence, persistence and incidence rates for specific HPV genotypes were also calculated.

We undertook an adjusted logistic regression to identify factors associated with persistent HPV, adjusting for repeated measures from individuals. Variables included in the model were demographic (current/past smoking, duration of HIV infection, current viral load and CD4 count, past tonsillectomy) and sexual behaviors (condom use during oral sex, ejaculation into mouth, tongue-kissing, receptive oral sex, touching partner’s anus with mouth or tongue). After including all variables with p value <0.1, backward stepwise logistic regression provided a final model for HPV detection. Statistical significance was set as probability values of less than 0.05. This research was approved by the Alfred Health Human Ethics Committee (Project 384/12).

## Results

Of 249 HIV-positive MSM from the 2010 study, 210 (84%) attended the centre during the 8 months recruitment period. Thirty-seven men declined to participate (30 were too busy, 3 were sick on the day, 4 were not interested) and 173 (69%) men were recruited. The mean age of participants was 52 years (SD ±14). The average duration of HIV (from first positive Western blot test) was 11.8 years, the mean current CD4 count was 679 cells/µl and 91% of participants had a suppressed viral load (<20 copies/mL). Ninety-four % were currently on antiretrovirals and 32% were current smokers.

Overall, beta globin, a measure of sample adequacy, was positive in 173 (100%) samples for ORS. The cell number estimation performed by comparison of crossing points from the real-time beta-globin PCR with known standards showed no difference between cell numbers obtained between those that were HPV positive and negative samples.

### Prevalence and incidence

Of the 173 men tested in 2013, 33 had at least one HPV genotype (19%, 95% CI: 14–26), 20 had at least one hr-HPV (12%, 95% CI: 7–17) and 7 had HPV 16 (4%, 95% CI: 2–8) detected. In 2010, 30 had at least one HPV genotype (17%, 95% CI: 12–23), 15 at least one hr-HPV (9%, 95% CI: 5–14) and 8 had HPV 16 (5%, 95% CI: 2–9) detected.

Twenty four men (14%, 95% CI: 9–19) had at least one incident HPV genotype in 2013. Fourteen men (8%, 95% CI: 5–13) had at least one incident hr-HPV. Of these, 4 had new HPV 16 detections. The incidence rate of detection of at least one new HPV genotype was 4.8 per 100 person years (95% CI: 3.1–7.0), of at least one hr-HPV genotype was 3.2 per 100 person years (95% CI: 1.8–5.1) and of HPV 16 was 0.8 per 100 person years (95% CI: 0.2–2.0).

The prevalence of specific genotypes is summarized in Table 1. There were 60 HPV genotype detections in 2013, of which 42 were incident detections. Of these, 25 were incident hr-HPV genotypes of which 4 were HPV 16.

**Table 1:** Frequency of HPV genotype detected in 2010 and 2013 samples with high risk HPV genotypes in bold.

HPV genotype	Baseline n	3 years latern (persisted from baseline)
6	1	1
11	2	2 (1)
**16**	**8**	**7 (4)**
**18**	**1**	**4**
**31**	**1**	**2 (1)**
**33**	**0**	**2**
**35**	**0**	**4**
**45**	**2**	**2 (1)**
**51**	**0**	**2**
**52**	**0**	**2**
53	2	1 (1)
55	6	2 (1)
**56**	**3**	**1**
**58**	**0**	**1**
**59**	**2**	**1**
61	2	0
62	4	7 (3)
**66**	**2**	**3**
69	3	1
70	1	2
72	8	4 (4)
73	2	1
82	0	3
83	1	1
84	1	2
89	0	2
Total any HPV	52	60 (16)
Total hr-HPV	19	31 (6)
Negative	139	136
Unable to type	4	3

### Persistence and clearance

To evaluate HPV persistence, we compared the 2010 HPV profiles of HPV-positive men with their 2013 HPV profile. Of 30 men in 2010 with any HPV genotype detected, 14 (47%, 95% CI: 28–63) had at least one persistent genotype. Of the 15 men in 2010 with hr-HPV detection, 6 men (40%, 95% CI: 16–68) had at least one persistent hr-HPV genotype. Of the 8 men in 2010 with HPV16 detection, 4 men (50%, 95% CI: 16–84) had persistent HPV16 detection. The clearance rate was 14.9 per 100 person years (95% CI: 8.2–24.2) for any HPV, 18.2 per 100 person years (95% CI: 8.2–32.7) for hr-HPV and 17.4 per 100 person years (95% CI: 5.0–38.8) for HPV 16.

There were 52 HPV genotype detections in 30 individuals at baseline (2010). Sixteen (31%, 95% CI: 19–45) genotypes were identified again in the same individuals in 2013 (Table 1). Of 19 hr-HPV detections in 2010, 6 (32%, 95% CI: 13–57) were identified again in 2013. Of 8 HPV16 detections, 4 (50%, 95% CI: 16–84) were identified again. The clearance rate per genotype detected was 23.3 per 100 HPV genotype years (95% CI: 16.7–29.8), 22.8 per 100 hr-HPV genotype years (95% CI: 12.7–35.8), and 16.7 per 100 HPV 16 genotype years.

### Factors associated with persistent detection

We found no sexual behavior variables were statistically associated with persistent oral HPV (any or high risk types). Longer duration of HIV (OR 1.13 (per additional year), 95% CI: 1.00–1.26) and past tonsillectomy (OR 8.17, 95% CI: 1.30–51.40) were the only two demographic factors associated with persistent HPV detection compared to men with non-persistent (any) HPV. These same factors were not statistically significant for hr-HPV, most likely due to the small sample numbers of hr-HPV in our sample. We did not find any statistically significant relationship between current CD4 count and HPV prevalence (p = 0.88) or persistence (p = 0.48).

## Discussion

This study extends the body of knowledge of the natural history of oral HPV in HIV-positive MSM by demonstrating that 43% of men had at least one of the same oral HPV genotypes and that 40% of men had a least one high-risk HPV genotype detected again three years later. In a comparable study of HIV-positive MSM, it was found that 52% of any oral HPV genotype persisted for a median of 2 years, however the study did not provide any clearance rate for hr-HPV [7]. This is quite different from HIV-negative men where the median duration of any oral HPV detection was 6.9 months (95% CI 6.2–9.3) and 6.3 months (6.0–9.9) for high-risk HPV [13]. Our findings indicate that persistence of hr-HPV and in particular of HPV-16 may be a concern in HIV-positive men for their future malignant potential. This may explain why people living with HIV has been shown to have an elevated risk of oropharyngeal cancer [14], particularly those who are infected with HPV-16 [4].

We found that at either time point, about 1 in 10 men had at least one hr-HPV detected and of particular concern is that of 8 HPV16 genotypes detected in 2010, 4 were still present in the same men three years later. Persistent infection is a risk factor for malignant transformation in genital epithelium and even though hr-HPV genotypes (especially HPV16) are consistently detected in the majority of oropharyngeal squamous cell cancers [4], the prognostic significance of oral detection of HPV genotypes that are high risk for cervical cancer is unclear. It will be important in future studies to determine if the development of oropharyngeal cancer is due to failure to clear hr-HPV genotypes or repeated exposure to hr-HPV over a prolonged period or both. More frequent oral HPV testing within the 3 years would have given a more accurate assessment of the evolution of HPV infection. This is an area that warrants further research.

The detection rates of 19% of participants with any oral HPV, 12% with hr-HPV, and 4% with HPV16 in a HIV-positive population stands in contrast to the US general population prevalence of 7% for any oral HPV, 4% for hr-HPV and 1% for HPV 16 [15]. Other studies confirm that HIV-positive persons are consistently at higher risk for oral HPV [6], [16]. Reasons for this may be due to differences in sexual behaviors [6] or that HIV itself may disrupt the mucosal epithelium of the mouth, thus facilitating penetration by HPV [17]. It has been demonstrated that co-infection with HIV may contribute to increased persistence or progression of oral HPV infection [18].

Whilst there are other published studies evaluating sexual behaviors with detection of any oral HPV [18], this is the first study to evaluate the role of sexual behaviors with persistent HPV detection in HIV-positive MSM. Of note, none of the sexual behaviors were associated with persistent HPV detection although the study had limited power to detect these. Duration of HIV infection has been shown to be associated with detection of oral HPV [6], [16] although this is the first study to demonstrate its association with *persistent* HPV. This may suggest that the underlying host immunity may be of greater importance than sexual behavioral factors for persistent oral HPV. The exact mechanism of this interaction between the duration of HIV and persistent oral HPV has been largely uninvestigated and warrants further research.

Increased association of tonsillectomy with persistent HPV detection has not been reported previously. Tonsils are an important secondary lymphoid organ in the immune system but tonsillectomy has not been shown to significantly impact humoral immune status in a HIV-negative population [19]. However the impact on mucosal immunity caused by tonsillectomy on persistent HPV detection in those living with HIV is unknown. This finding warrants further investigation to see if there is any clinical significance for the association between tonsillectomy and the development of oropharyngeal carcinoma in HIV-positive people.

Due to the limitations of small numbers of persistent HPV and issues around detecting true persistence in previous studies and this study, it remains unclear whether there are additional factors that may increase the likelihood of persistence of HPV. A larger study to identify these factors would help stratify those at increased risk for persistent hr-HPV and theoretically those at higher risk for oropharyngeal cancer.

Our study has a number of limitations that need to be considered. Firstly although we used the same method for oral sampling that others have used [6], [18], it is possible that this method misses some infections. If this is the case, we will have overestimated clearance and underestimated persistence of infection. In our study, we have tried to minimize missed infections by using two different methods including a very sensitive SPF10-LiPA genotype assay. Unfortunately there is not a more sensitive collection method currently available that has been widely used. Secondly, we only sampled individuals at two time points which meant that we, like similar natural history studies, may call HPV infection persistent when it results from transient exposure to the same genotype prior to the two occasions. It is possible that it may be detection from the same regular partner that was present at both times and given we did not collect this information we cannot rule out this explanation. Finally, due to small sample sizes, we had limited statistical power to detect risks for persistence.

## Conclusion

Nearly half of oral HPV genotypes, detected in HIV-positive men who have sex with men, were detected again 3 years later. Persistent HPV detection was associated with duration of HIV infection and tonsillectomy.

## Supporting Information

Appendix S1 **Questionnaire.** Written questionnaire completed by participants.
(DOC)
