# Supplementary material for: Detection of Oral Human Papillomavirus in HIV-Positive Men Who Have Sex with Men 3 Years after Baseline: A Follow Up Cross-Sectional Study
Source: PLoS One. 2014 Jul 17;9(7):e102138. doi: 10.1371/journal.pone.0102138 (PMC4102527; doi:10.1371/journal.pone.0102138)
Supplement: Appendix S1 — Questionnaire. Written questionnaire completed by participants. (DOC) [file pone.0102138.s001.doc]

**Appendix S1 Questionnaire completed by participants**

**DOB:**

**Date:**

**Oral Human Papillomavirus (Follow up) Project**

**Confidential Questionnaire**

## Questions about things that might affect your mouth and throat.

**1. How many hours since you last brushed your teeth?_____________**hours.

**2. How many times a day do you usually brush your teeth?**_________times per day.

**3. How many hours since you flossed your teeth?**

__________hours ago

OR ________days ago

OR ⬜ if more than 1 week ago

OR ⬜ never flossed

**4. Do you use mouthwash more than once a week?**

⬜ Yes

⬜ No

**5. Were you smoking when you last provided your sample in April 2010.**

⬜ No - GO TO Q6

⬜ Yes – GO TO Q7

**6. Did you start smoking since your last sample (April 2010)?**

Yes. When did you start smoking?_____________(month/year)

No

**7. Are you still smoking currently or did you quit?**

Still smoking. How many cigarettes a day do you smoke? _________________

Quit. When did you stop? __________________(month/year)

**8. Have you had a tonsillectomy (ie. removal of tonsils)?**

Yes

No

## Questions about Human Papillomavirus (HPV)

**9. Since you last provided a sample (April 2010) have you had warts on your face, mouth or tongue?**

Yes

No – GO TO Q11

**10. Do you have warts on your face, mouth or tongue now?**

Yes

No. How long ago did you have warts on your face, mouth or tongue? ...........years. (*Write <1 if the warts disappeared or were removed less than one year ago)*

**11. *Since you last provided a sample (April 2010) have you* had warts on or near your penis or anus?**

Yes

No – GO TO Q13

**12. Do you have warts on or near your penis or anus now?**

Yes

No. How long ago did you have warts on or near your penis or anus? ...........years. (*Write <1 if the warts disappeared or were removed less than one year ago)*

## Questions about sex that might transmit HPV.

*For these questions, estimates are fine. No need to spend time remembering exact numbers.*

**13. How many days since you had sex with someone and:**

Had their tongue in your mouth . .......days, or  more than two weeks or  never

Had their penis in your mouth . ...... days, or  more than two weeks or  never

Rimmed their anus. (Touched their arse with your tongue or mouth)

...... days, or  more than two weeks or  never

Had their fingers in your mouth ...... days, or  more than two weeks or  never

Had anal sex (your penis in his anus).. days, or  more than two weeks or  never

Had anal sex (his penis in your anus).. days, or  more than two weeks or  never

**14. In the last two weeks, with how many people have you:**

Had their tongue in your mouth ________ people or  never

Had their penis in your mouth ________ people or  never

Rimmed their anus. (Touched their arse with your tongue or mouth)

________ people or  never

Had their fingers in your mouth ________ people or  never

Had anal sex (your penis in his anus) ________ people or  never

Had anal sex (his penis in your anus) ________ people or  never

**15. In the last year, with how many people have you:**

Had their tongue in your mouth ________ people or  never

Had their penis in your mouth ________ people or  never

Rimmed their anus. (Touched their arse with your tongue or mouth)

________ people or  never

Had their fingers in your mouth ________ people or  never

Had anal sex (your penis in his anus) ________ people or  never

Had anal sex (his penis in your anus) ________ people or  never

**16. Since your last visit (April 2010). with roughly how many people have you:**

Had their tongue in your mouth ________ people or  never

Had their penis in your mouth ________ people or  never

Rimmed their anus. (Touched their arse with your tongue or mouth)

________ people or  never

Had their fingers in your mouth ________ people or  never

Had anal sex (your penis in his anus) ________ people or  never

Had anal sex (his penis in your anus) ________ people or  never

**17. Of all the men you have given oral sex (their penis in your mouth), how often did these men wear a condom during oral sex with you?**

Half or more

Only some

None or hardly any

**18. Of all the men you have given oral sex (their penis in your mouth), with how many have you continued until they come (ejaculate)?**

Half or more

Only some

None or hardly any

Thanks for completing this.
